# Is *FMR1* CGG Repeat Number Polymorphism Associated With Phenotypic Variation in the General Population? Report From a Cohort of 5,499 Adults

**DOI:** 10.3389/fpsyt.2021.727085

**Published:** 2021-08-11

**Authors:** Jinkuk Hong, Leann DaWalt, Mei Wang Baker, Elizabeth M. Berry-Kravis, Marsha R. Mailick

**Affiliations:** ^1^Waisman Center, University of Wisconsin-Madison, Madison, WI, United States; ^2^Wisconsin State Laboratory of Hygiene, Madison, WI, United States; ^3^Departments of Pediatrics, Neurological Sciences, Biochemistry, Rush University Medical Center, Chicago, IL, United States

**Keywords:** *FMR1* CGG repeats, genotype-phenotype associations, genetic epidemiology, population cohorts, normal genetic variation

## Abstract

*FMR1* CGG repeat length was assayed in 5499 research participants (2637 men and 2862 women) in the Wisconsin Longitudinal Study (WLS), a population-based cohort. Most past research has focused on clinically-ascertained individuals with expansions in CGG repeats, either those with fragile X syndrome (> 200 CGG repeats), the *FMR1* premutation (55–200 repeats), or in the gray zone (variously defined as 45–54 or 41–54 repeats). In contrast, the WLS is a unique source of data that was obtained from an unselected cohort of individuals from the general population for whom *FMR1* CGG repeat length was assayed. The WLS is a random sample of one-third of all high school seniors in the state of Wisconsin in 1957. The most recent round of data collection was in 2011; thus, the study spanned over 50 years. Saliva samples were obtained from 69% of surviving members of the cohort in 2008 and 2011, from which CGG repeats were assayed. With one exception, the CGG repeat length of all members of this cohort was below 100 (ranging from 7 to 84). The present study evaluated the genotype-phenotype associations of CGG repeat number and IQ, college graduation, age at menopause, number of biological children, having a child with intellectual or developmental disabilities, and the likelihood of experiencing an episode of depression during adulthood. Linear and curvilinear effects were probed. Although effect sizes were small, significant associations were found between CGG repeat length and high school IQ score, college graduation, number of biological children, age at menopause, and the likelihood of having an episode of depression. However, there was no significant association between repeat length and having a child diagnosed with an IDD condition. This study demonstrates a continuum of phenotype effects with *FMR1* repeat lengths and illustrates how research inspired by a rare genetic condition (such as fragile X syndrome) can be used to probe genotype-phenotype associations in the general population.

## Introduction

The *FMR1* gene on the X chromosome plays a critically important role in the development and functioning of the nervous system, as its protein product normally regulates the translation of ~30% of all transcripts in the pre- and post-synaptic proteomes critical for synaptic plasticity (1). Expansions above a critical threshold of a CGG triplet repeat (i.e., 200 CGG repeats) in the 5′ untranslated region cause fragile X syndrome (FXS), the most common inherited form of intellectual disability and autism. Additionally, repeats in the premutation range (55–200 repeats) and to a lesser extent in the gray zone (variously defined as 45–54 or 41–54 repeats) have received research attention leading to observation of phenotypic associations (2, 3).

However, the number of CGG repeats in *FMR1* is highly polymorphic in the human population (4, 5). The peak value for CGG repeats is at 30, with >90% of individuals having fewer than 40 and the lowest being 6 repeats (4, 6–8). In 2003, Chen et al. (7) reported the results from studies of synthetic human *FMR1* promoter sequences driving a luciferase reporter in transfected cell lines, showing that CGG repeats had no significant effect on transcription. However, compared to the modal number of approximately 30 CGGs, both lower and higher numbers of CGG repeats negatively affected translation of luciferase mRNA into protein, suggesting that the modal number of 30 may maximize translational efficiency. This observation predicts that repeats across the CGG range, above and below 30, may be associated with varying degrees in the efficiency of translation of the *FMR1* transcript, and thus may be associated with phenotypic variability. Wang et al. (9) have suggested that even subtle changes in both protein and mRNA levels could have wide-ranging effects both on brain structure and working memory in healthy adult men with normal *FMR1* alleles.

Most studies of genotype-phenotype associations in *FMR1* are based on data derived from clinical populations. Following diagnosis of a child with FXS, the family may be tested to determine if each member has expansions in the number of CGG repeats or is in the normal range. Thus, the majority of variation in CGG repeat number is not evaluated in these studies because only those at the very high end of the repeat range, and their relatives, are generally assayed. Understanding the true significance of the variations across much of the yet-unstudied range of *FMR1* CGG repeats would be advanced by study of a population-based sample.

The Wisconsin Longitudinal Study (WLS) is a unique source of phenotypic data that has been genotyped for CGG repeats in *FMR1*. It is a random sample of a cohort (mostly born in 1939), initially studied as high school seniors in 1957 and subsequently studied periodically through age 71 (10). Capitalizing on the availability of DNA isolated from saliva samples collected in 2008 and 2011, the *FMR1* CGG repeat number of the members of this cohort was ascertained, enabling study of genotype-phenotype associations across much of the CGG repeat range in a population-based sample of adults.

## Method

### The Present Study

The key question asked in the present study was, to what extent is polymorphism in *FMR1* CGG repeat number (which in this analysis ranged from 7 to 84 repeats) associated with individual variation in phenotypic characteristics? Although there have been previous studies of genotype-phenotype associations using *FMR1* CGG repeat data derived from participants in the WLS, there are three major differences between the previously reported WLS studies and the present research.

First, all past studies of genotype-phenotype associations in WLS included both the original cohort of Wisconsin 1957 high school graduates (referred to as the “graduates”) as well as a sub-set of their siblings. However, in the present study, we analyze data from the graduate cohort only, not including the sibling participants. Whereas the majority of the members of the original cohort of 1957 high school graduates were born in 1939, their siblings who participated in the WLS (including step- and half-siblings) spanned the birth years of 1906 to 1970. Because some of the key phenotypes analyzed for the present study may have reflected societal trends that change over time (e.g., college attendance, number of children), we focused on an age cohort (i.e., 1957 high school graduates) to control for the influence of these secular trends.

Second, all prior WLS reports that analyzed *FMR1* CGG repeat data included only those participants who provided saliva samples in 2008 (*n* = 4,382 graduates provided samples). In 2011, an additional 1,118 graduates who had not responded to the original request provided such samples. Together, the samples obtained in 2008 and 2011 (*n* = 5,500) constituted DNA from 69% of the surviving members of the original cohort of high school graduates, all of which were assayed for *FMR1* CGG repeats. The present study's analysis of genotype-phenotype associations is the first to include data from all participants assayed for *FMR1* CGG repeats. However, for reasons described below, one case was dropped from the present analysis, resulting in an analytic sample of 5,499 adults.

Lastly, the statistical approach used in our past studies contrasted specific clinically-defined segments of the CGG repeat distribution (e.g., premutation vs. controls) (11), contrasted statistically-defined segments of the repeat distribution (e.g., “low zone” vs. controls) (12), or evaluated interaction effects between repeat number and environmental factors (e.g., parenting a child with a disability) (13). In contrast, the present analysis treated CGG repeats as a continuous variable and directly probed genotype-phenotype associations. Thus, the central question of the present study was whether *FMR1* CGG repeat number is associated with phenotypic characteristics in the general population, not in the context of stress exposure or in clinically- or statistically-defined segments of the repeat distribution.

### Hypotheses

The hypotheses advanced in this study were based on past research examining three specific phenotypes that have been implicated in past research on *FMR1* CGG repeats below the full mutation—cognitive, reproductive, and psychiatric phenotypes. However, most past research has focused on the upper end of the repeat range (i.e., gray zone and premutation expansions), with only a small number of studies extending across the full CGG repeat range (13–16) or specifically probing the low end of the repeat distribution (12). Although we predicted directional associations between *FMR1* CGG repeat number and specific phenotypic characteristics, the study was exploratory as, to the best of our knowledge, no past research has been conducted on a large unselected population-based cohort without clinical ascertainment of cases. Small effect sizes were expected.

Regarding the cognitive phenotype, we hypothesized that there would be a negative association between CGG repeat number and *IQ score*, based on past research showing that men and women who had greater numbers of CGG repeats have subtle limitations in cognitive functioning, including executive functioning limitations (3, 17, 18). We further expected a similar association for achieving a *college degree*, although this hypothesis was tested for men only, for reasons explained below.

Regarding the reproductive phenotype, the primary measure of women's fertility was *age at menopause*. Additionally, we evaluated the *number of biological children* born to women. We predicted that there would be negative associations between CGG repeat number and age at menopause, and also with number of biological children, based on past research that reported early menopause and infertility in women with CGG expansions in the premutation range (19–23). We also explored whether women at the higher end of the CGG distribution would be more likely to have *a child with a developmental disability* (24).

Regarding the psychiatric phenotype, past research reported higher rates of *depression* in men and women who had a higher number of CGG repeats (3, 21, 25). Therefore, we expected a positive association between CGG repeats and experiencing an episode of depression.

Much past research on genotype-phenotype associations in *FMR1* reported curvilinear associations, particularly for fertility-related and psychiatric phenotypes (13, 15, 19, 26, 27). In contrast, phenotypes associated with FXTAS (Fragile X-associated Tremor /Ataxia Syndrome) have shown a linear association between repeat number and symptoms (28, 29). Therefore, both linear and curvilinear associations were probed in this study.

### Study Population and Data

Data for the present study were drawn from the WLS, a public use data set. It consists of a random sample of 10,317 women and men who graduated from Wisconsin high schools in 1957, representing one-third of that cohort (10). In 1957, 75% of Wisconsin residents who were of high school age graduated from high school. Follow-up studies were conducted in 1975 with 9138 members of the original cohort when they were, on average, 36-years old; in 1992 with 8493 respondents when they were in their early 50s; in 2004 with 7265 respondents when they were in their mid-60s; and again in 2011 with 5967 respondents when they were in their early 70s. The participants in the 2011 study constituted 72.2% of the surviving members of the original cohort.

Although all of the original WLS participants were high school graduates, these WLS participants ranged in IQ score from a low of 61 to a high of 145. Fully 15% had IQ scores of 85 (one SD below the mean) or below. This percentage is nearly the expected proportion of the population on the low end of the IQ distribution (16% of the population is expected to be one SD below the mean or lower). The inclusion of individuals with lower IQs in the WLS population is an important sample characteristic, given past research suggesting a possible cognitive phenotype of the premutation of the *FMR1* gene. Reflecting Wisconsin's population in the mid-20^th^ century, the WLS sample is racially and ethnically homogeneous; 99.2% are White and the majority (84.2%) are of Northern European heritage.

In 2008 and 2011, WLS collected saliva samples from participants using Oragene kits (DNA Genotek, Inc., Bethlehem, PA). All participants provided informed consent under a protocol approved by the Institutional Review Board of the University of Wisconsin-Madison. More than two-thirds (69.0%) of surviving WLS members provided saliva samples, and analysis of these samples constitute the basis of the research presented here. Those who provided saliva samples had one-half year more schooling (13.8 years vs. 13.3 years, *p* < 0.001) and three points higher IQ scores (102.2 vs. 98.4, *p* < 0.001) than those who did not return saliva samples. Otherwise, they were representative of the WLS graduate sample as a whole.

### Determination of the *FMR1* CGG Triplet Repeat Number

The present study includes 5499 WLS participants (2637 men and 2862 women) for whom saliva samples were obtained and *FMR1* CGG repeats were assayed. DNA was isolated using standard methods. For saliva samples collected in 2008, the number of *FMR1* CGG repeats was determined (under the supervision of author MWB) using a PCR-based protocol that incorporated reagents developed and manufactured by Celera Corporation (Alameda, CA). See Seltzer et al. (11) for details. Additional participants provided saliva samples in 2011; for these samples, repeat number was determined via an assay using the Asuragen AmplideX® Kit (30, 31), conducted in the Rush University Medical Center Molecular Diagnostics Laboratory (supervised by author EB-K). A concordance study was conducted between the two assays. DNA samples initially collected and assayed in 2008 (*n* = 22; some from the premutation range and some with normal alleles) were re-assayed using the Asuragen assay. The correlation between the two was .9996.

For the women, the assays yielded CGG repeat data on the *FMR1* gene on both X chromosomes. Because we did not have activation ratio data, one X chromosome was selected for analysis in the present study as follows. Although we considered alternative approaches (32, 33), we followed the approach of Hunter and colleagues (34). We selected the *longer* allele in women who had one expanded (i.e., > 40 CGGs) and one normal allele (*n* = 194) and in the four cases who had two expanded alleles. Similarly, we selected the *shorter* allele in women who had one low allele (i.e., <26 CGGs) and one normal allele (*n* = 878) and in women who had two low alleles (n = 139). We randomly selected one allele for analysis in the present study in women who had two normal alleles (between 26 and 40 CGG repeats, *n* = 1589), and also for those with one low allele and one expanded allele (*n* = 58).

### Measurement of Phenotypic Characteristics

All rounds of data collection from the WLS were used to measure the phenotypes evaluated in the present study. In 1957, the source of data was high school records. In 1975, data were collected via a telephone interview. In the 1992 and 2004 rounds of data collection, data were collected via a telephone interview and self-administered questionnaires. In 2011, data were collected via an in-person home visit and self-administered questionnaires. For the present analysis, we evaluated the association between *FMR1* CGG repeat number and indicators of the cognitive, reproductive, and psychiatric phenotypes implicated in prior *FMR1* research.

#### Cognitive Phenotype

Two measures of cognitive functioning were analyzed. The Henmon-Nelson Test of Mental Abilities (35), was administered when participants were high school students. *IQ scores* were obtained by the WLS from high school records. As noted, participants' scores ranged from 61 (the floor of the test) to 145. See Maenner et al. (36) for details regarding the measurement of IQ in the WLS.

A second measure of cognitive functioning was included, namely *attainment of a college degree*. As all WLS participants included in this study were high school graduates, the measure of educational attainment analyzed for the current research was college graduation. Although most of those who obtained a college degree did so by the 1975 round of data collection, a small number of participants did so subsequently, and thus those who obtained a college degree at any point up to the 2011 round of data collection were included in this analysis.

#### Reproductive Phenotype

The primary measure of women's fertility was self-reported *age at menopause*. Additionally, we evaluated the *number of biological children born to women*. Women whose ovaries or uterus had been surgically removed (*n* = 1,048) were not included in the analysis of age at menopause. Additionally, among women who had at least one biological child, we used a dichotomous variable indicating whether any of their children had been diagnosed with an *intellectual or developmental disability* (IDD). See Mailick et al. (13) for details describing the determination of disability status in WLS children. All of these measures were obtained in the 2004 round of WLS data collection, when women were age 65.

#### Psychiatric Phenotype

Symptoms of *depression* were assessed by the Center for Epidemiological Studies-Depression Scale (CES-D) (37), administered at three rounds of WLS data collection (1992, 2004, and 2011 when participants were ages 53, 65, and 72, respectively). For each of 20 depression symptoms, the participant was asked to indicate how many days in the past week the symptom was experienced (0 = never to 3 = 5–7 days; α = 0.85). A score of 16 or above was considered an indicator of clinical depression (38, 39). We created a dichotomous variable indicating whether the participant had experienced an episode of clinical depression (i.e., a score of 16 or higher on the CES-D) at any point of data collection.

### Data Analysis

CGG repeats in the present cohort ranged from 7 to 128. The next highest number of repeats was 84. Although the case with 128 repeats did not qualify as a statistical outlier as estimated with Cook's Distance (Cook's D), inclusion of the case inflated the standard deviation of the CGG repeat range variable, leading to less precise estimates from the regression models. Therefore, it was decided to focus the present analysis on participants with 84 or fewer CGG repeats. The alpha level was set at .05.

Means and standard deviations of study variables are presented in Table 1. Data for college graduation are presented for men only because, whereas men and women had identical IQ metrics, substantially fewer women than men attended college (23.9% vs. 34.2%, chi square = 69.8, *p* < 0.001), reflecting sex-specific differences in expectations for college education in 1957. Thus, college graduation was not considered to be a valid measure of cognitive ability for this cohort of women. Data for age at menopause, number of biological children, and whether any child had an IDD diagnosis are presented for women only, given past studies of the association between *FMR1* CGG repeat number and these characteristics, as cited above.

**Table 1:** Descriptive statistics of study variables^a^.

	**Male**	**Female**	**Total**
CGG repeats	30.1 (5.4) [9, 65] (*n* = 2,637)	selected allele: 29.2 (6.8) [7, 84] (*n* = 2,862)	29.7 (6.2) [7, 84] (*n* = 5,499)
		long allele: 32.8 (5.0) [21, 84]	
		short allele: 27.5 (4.4) [7, 43]	
Birth year	1939.3 (0.52) [1936, 1941] (*n* = 2,622)	1939.5 (0.48) [1930, 1940] (*n* = 2,850)	1939.4 (0.50) [1930, 1941] (*n* = 5,472)
IQ	102.1 (15.2) [61, 145] (*n* = 2,637)	102.3 (14.3) [61, 145] (*n* = 2,862)	102.2 (14.8) [61, 145] (*n* = 5,499)
% college graduate	34.2% (*n* = 2,619)	–	–
Age at menopause	–	50.8 (4.7) [20, 65] (*n* = 1,448)	–
Number of biological children	–	2.84 (1.6) [0, 11] (*n* = 2,703)	–
% having a child with IDD	–	1.4% (*n* = 2,438)	–
% having episodes of clinical depression	17.0% (*n* = 2,517)	24.2% (*n* = 2,773)	20.8% (*n* = 5,290)

a *Means, standard deviations (in parentheses), and range (in brackets) are presented unless the variable is marked with (%)*.

The analytic approach used in this paper treated CGG repeat number as a continuous variable. For the multivariate analyses, Ordinary Least Squares (OLS) and logistic regressions were conducted. For sex-specific analyses (college graduation, age at menopause, number of biological children, having a child with IDD), the regression models included two stages. Model 1 included birth year of the participant as a control variable and CGG repeat number. In Model 2, the squared term of CGG repeats was entered to evaluate curvilinear associations with the dependent variable. For the analysis of IQ and episodes of depression, the regression models included four steps: Models 1 and 2 were the same as above, with the addition of sex of the participant. In Models 3 and 4, interaction terms (CGG repeat X sex, CGG repeat squared X sex) were added to the regression models to evaluate whether the CGG effect differed for men and women. For all analyses, significant CGG effects were graphed showing the scatter plots and the line of best fit between CGG repeat number and the phenotypic variable.

## Results

### Descriptive Findings

**Figure 1:**
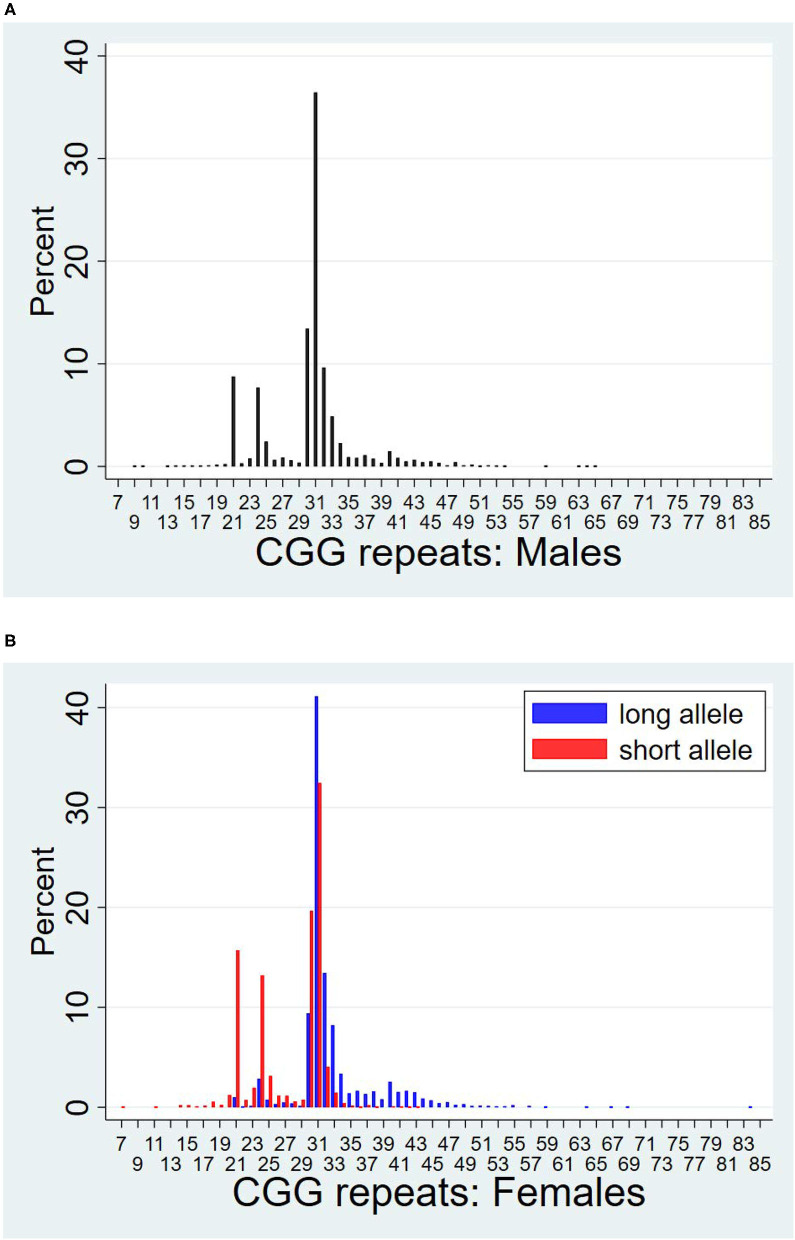
Distributions of CGG repeat lengths by sex. **(A)** Males. **(B)** Females.

Figure 1 presents a histogram of the distribution of CGG repeats for men (Figure 1A) and women (Figure 1B). For women, the histogram includes the distributions of both the long and short allele. Women's repeat length on their shorter and longer alleles are also reported in Table 1. As shown in Table 1, the mean number of repeats for the cohort was 29.7, ranging from 7 to 84 repeats, with men averaging approximately one more repeat than women (30.1 vs. 29.2, *p* < 0.001).

Table 1 also reports the average birth year of study participants, with 1939 being the mean for both men and women. Descriptively, 77% of participants were born in 1939, 12% in 1938, and 9% in 1940. The remaining participants (*n* = 5) were born in earlier or later years.

IQ scores were nearly identical for men and women, averaging 102.2. One-third of the men achieved a college degree. Women averaged 2.84 biological children, ranging from 0 to 11 children. Women who had a natural menopause (i.e., not having surgical removal of ovaries or uterus) averaged 50.7 years of age when they last menstruated (ranging from 20 to 65 years of age). Of women who had at least one biological child, very few (1.4%) reported having a child with a diagnosis of IDD. Women and men differed in their likelihood of having an episode of depression at one or more of the points of data collection when the CES-D was administered, with women being significantly more likely than men (24.2 vs. 17.0%, p < 0.001).

### Multivariate Findings

Full regression models are presented in Table 2. In preliminary analyses of the models including both men and women (IQ, experiencing an episode of depression), terms evaluating the interaction effects of CGG (and CGG squared) with sex were tested. However, neither of these interaction terms reached statistical significance and therefore are not reported in Table 2.

**Table 2:** Associations between CGG repeat length and phenotypes.

	**Model 1:**	**Model 2:**
**A. IQ^a^**		
Birth year	6.55 (0.39)^***^	6.55 (0.39)^***^
Sex (female = 1)	−0.707 (0.394)	−0.717 (0.398)
CGG	−0.069 (0.031)^*^	−0.072 (0.034)^*^
CGG-squared	–	0.0004 (0.0023)
**B. College graduate—males^b^**		
Birth year	2.24^***^ [1.87, 2.68]	2.25^***^ [1.88, 2.69]
CGG	0.985^*^ [0.969, 0.999]	0.982^*^ [0.967, 0.998]
CGG-squared	–	1.000 [0.999, 1.002]
**C. Age at menopause—females^a^**		
Birth year	−0.468 (0.278)	−0.459 (0.276)
CGG	−0.014 (0.019)	0.019 (0.021)
CGG-squared	–	−0.004 (0.001)^***^
**D. Number of biological children—females^a^**		
Birth year	−0.060 (0.066)	−0.061 (0.066)
CGG	−0.000 (0.005)	−0.006 (0.005)
CGG-squared	–	0.0010 (0.0003)^**^
**E. Having a child with Intellectual or developmental disability—females^b^**		
Birth year	0.67 [0.45, 1.00]	0.67 [0.45, 1.00]
CGG	0.981 [0.930, 1.033]	0.978 [0.928, 1.031]
CGG-squared	–	1.001 [0.997, 1.003]
**F. Episodes of clinical depression^b^**		
Birth year	0.79^***^ [0.69, 0.90]	0.79^***^ [0.69, 0.90]
Sex (female = 1)	1.59^***^ [1.39, 1.83]	1.60^***^ [1.39, 1.84]
CGG	0.988* [0.977, 0.998]	0.989 [0.978, 1.000]
CGG-squared	–	1.000 [0.999, 1.001]

* *p < 0.05;*

** *p < 0.01;*

*** *p < 0.001*.

a *Unstandardized coefficients are presented with standard errors in parenthesis*.

b *Odds ratios are presented with 95% confidence intervals in brackets*.

#### Cognitive Phenotype

**Figure 2:**
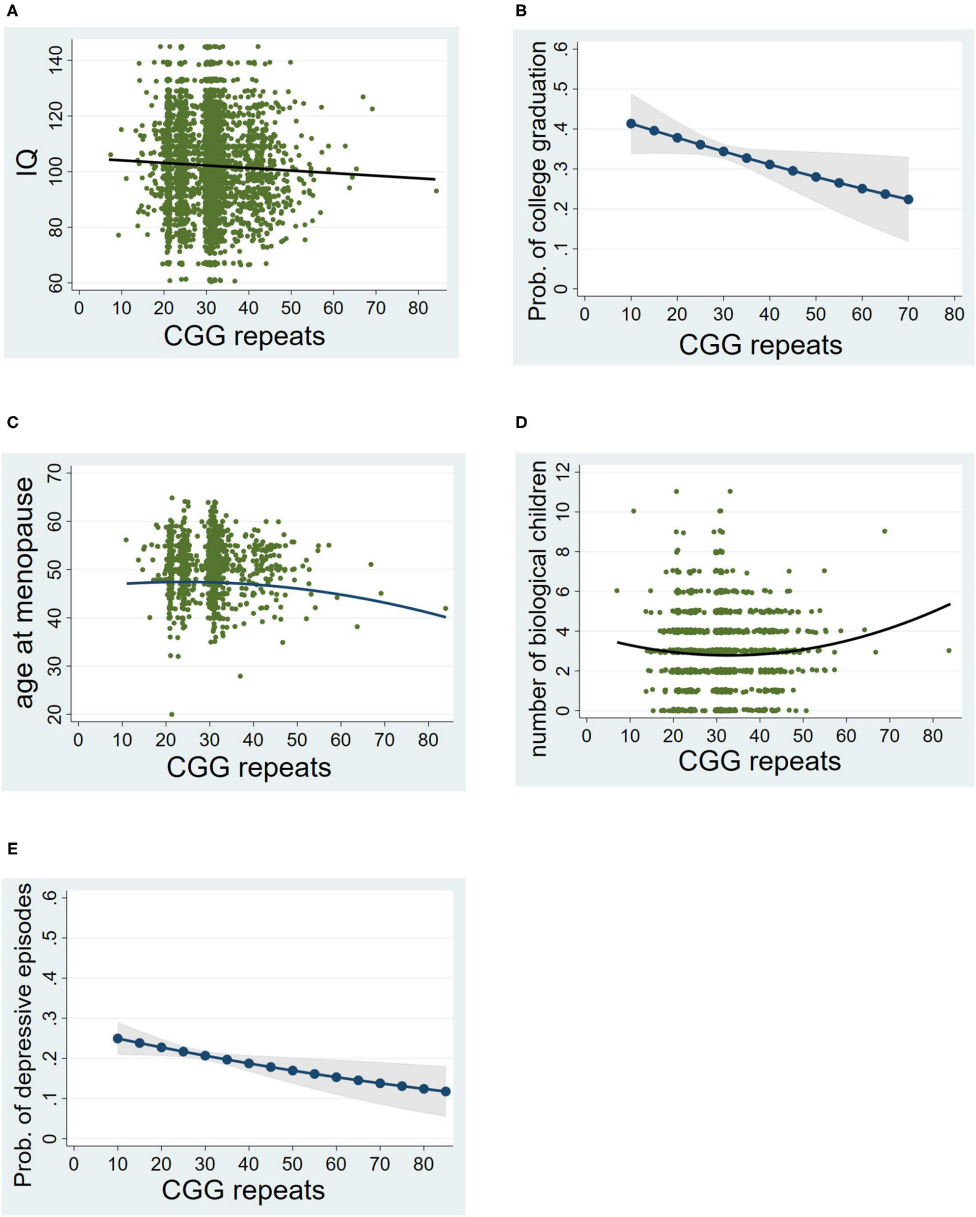
Associations between CGG repeat lengths and phenotypes. **(A)** Linear association with IQ. **(B)** Linear association with college graduate (males only). **(C)** Curvilinear association with age at menopause (females only). **(D)** Curvilinear association with the number of biological children (females only). **(E)** Linear association with episodes of clinical depression. For **(A)**, **(C)**, and **(D)**, a scatterplot with the best fitted line are shown. For **(B)** and **(E)**, predicted probability of the phenotype by CGG repeat lengths are presented; Prob, predicted probability.

As hypothesized, there was a significant negative linear association between CGG repeats and IQ score (see Table 2A). Figure 2A shows that those who had CGGs at the lower end of the repeat range had IQ scores just above the mean of 100 while those at the higher end of the repeat range scored below the mean. The CGG squared term was not significant.

A similar pattern was observed for college graduation rates among the men, as hypothesized (see Table 2B). As shown in Figure 2B, the probability of college graduation decreased as CGG repeats increased. Descriptively, nearly 40% of men in the lower end of the CGG distribution were college graduates, whereas at the upper end of the CGG distribution about 25% were college graduates.

#### Reproductive Phenotype

For the analysis of age at menopause, only women who went through menopause naturally (*n* = 1450) were included (excluding those whose uterus or ovaries were surgically removed). There was a significant curvilinear association between number of CGG repeats and age at menopause (see Table 2C). The shape of this association was consistent with the hypothesis. As shown in Figure 2C, women at the higher end of the CGG repeat distribution had an earlier age at menopause than those in the middle or lower end of the CGG repeat distribution. Notably, there was no significant association between CGG repeats and the likelihood of having surgical removal of the uterus or ovaries (*p* > 0.56).

There was a significant curvilinear association between CGG repeats and the number of biological children born to the women in the cohort (see Table 2D). All women were included in this analysis (*n* = 2,703), regardless of whether they went through menopause naturally or due to surgical removal of their uterus or ovaries. Unexpectedly, a *greater* number of children were born to those at the higher end of the CGG repeat distribution than those who were in the middle or lower end of the distribution (see Figure 2D), which was counter to our hypothesis. This effect remained significant even after controlling for social factors that might account for larger family size, such as number of marriages and Catholic religious affiliation. Both of these social factors were significant predictors of number of biological children (*p* < 0.001), and the CGG effect nevertheless remained statistically significant even with these factors controlled [regression coefficient (b) = 0.0009, standard error (s.e.) = 0.0003, *p* = 0.005].

In a sensitivity analysis, the association between CGG repeats and number of biological children was re-estimated for the sub-set of women included in the menopause analysis (i.e., those mothers whose menopause was not induced by surgical removal of the uterus or ovaries). Although this sub-set of women was only half as large as those included in the full analysis of number of biological children reported above (*n* = 1,402 vs. *n* = 2,703), the results were similar [b = 0.007, s.e. = 0.004, *p* = 0.057].

The association between CGG repeat number and whether women had a child with IDD was not statistically significant (see Table 2E).

#### Psychiatric Phenotype

There was a significant negative association between repeat number and the likelihood of exceeding a CES-D score of 16 at any of the three time points of measurement of depression (see Table 2F), which was counter to the hypothesis. As descriptively illustrated in Figure 2E, more than 20% of those at the lower end of the repeat distribution had at least one episode of depression during adulthood, while just over 10% of those at the upper end of the repeat range had at least one such episode.

## Discussion

To the best of our knowledge, the Wisconsin Longitudinal Study offers the first opportunity to evaluate genotype-phenotype associations in a large randomly selected cohort of the U.S. general population, without clinical ascertainment of any cases. The phenotypes evaluated were measures of cognitive functioning, reproductive characteristics, and a psychiatric condition, all selected based on past research on the phenotypes associated with *FMR1* CGG repeat number. There were significant genotype-phenotype associations, but the effects were small in magnitude. The effect of CGG repeats in the range evaluated in this study perhaps is best interpreted as contributing to non-clinical variation in cognitive, reproductive, and psychiatric characteristics in the general population rather than signifying clinical impairment. Nevertheless, understanding how polymorphisms in the *FMR1* gene contribute to “normal” phenotypic variation represents a contribution of the present research. More generally, the inclusion of genetic data in other large NIH-funded population survey studies, such as the MIDUS study (http://midus.wisc.edu/index.php) and Add Health (https://addhealth.cpc.unc.edu/), offers other opportunities for probing such effects of genetic variants.

In some respects, the associations observed in this study were consistent with genotype-phenotype correlations reported in clinical literature on the *FMR1* gene. For example, regarding cognitive functioning, higher numbers of CGG repeats were associated with somewhat lower IQ scores and less likelihood of achieving a college degree (by men), a pattern that points to the more substantial cognitive challenges observed in premutation carriers. Consistent with this observation, in a study of males with normal *FMR1* alleles, the influence of the gene on brain structure and working memory was established, suggesting that the *FMR1* gene is a genetic factor that has implications for the transmission of intelligence even within those who have normal alleles (9). Similarly, the earlier age at menopause among women who had higher numbers of CGG repeats is consistent with much past premutation research (19).

However, other associations were counter to the prediction. Although we hypothesized that women with higher numbers of repeats would report having *fewer* biological children, there was a positive association between repeat number and family size. Follow-up examination of the women in the premutation range in the present cohort who gave birth to their last child after age 30 revealed that they had a larger gap between their last two children (mean = 7.0 years) than other women in the WLS who gave birth to their last child after age 30 (mean gap = 5.0 years). It has been reported that women in the premutation range occasionally move in and out of menopause (19, 40), and it is possible that additional children could have been conceived during the period of skipped cycles. Additionally, as Allen et al. (19) noted, women in the low premutation range in their study (59–79 CGG repeats) were not different from non-carriers with regard to fertility problems or times to first pregnancy. Thus, the patterns observed here, mainly with respect to women in the low premutation range, are not inconsistent with past observations. Future research is needed to parse these effects. There was no association in the present study between repeat number and having a child with an IDD diagnosis, also possibly due to the restriction in the range of CGG repeats at the upper end of the distribution. It is further possible that the WLS population, limited to those who graduated high school, under-represented individuals likely to later give birth to a child with an IDD condition. This might account for the low rate of such children observed here.

The *inverse* association between CGG repeat number and probability of having an episode of depression also was counter to our prediction. Most past studies of the association between CGG repeats and depression were conducted on premutation carrier mothers of children with FXS who had CGG repeats in the upper premutation range or were premutation carriers with FXTAS (3, 25, 41). In these past studies, higher numbers of CGG repeats were associated with depression. In contrast, in the cohort analyzed for the present study, very few participants had children with IDDs and no participant had CGG repeats in the upper premutation range. Nevertheless, the direction of the relationship observed here between CGG repeats and episodes of depression was unexpected, and warrants evaluation in future population research.

The present study is not without limitations. CGG repeats in the present study were below 85 repeats, constraining the detection of phenotypes that could be associated with genotypic variation. Additionally, the WLS cohort lacks racial diversity and thus cannot be generalized to contemporary populations in the U.S. The cohort studied (mainly born in 1939, reaching adulthood in the late 1950s, preceding the baby-boom generation by 6 years) reflects secular trends of that time and as such is not representative of cohorts born in more recent years. Future research is needed to separate secular trends from genetic influences. The lack of measures of AGGs, activation ratio, and FMRP limits the extent of our understanding of genotype-phenotype associations. The findings that were counter to the hypotheses (regarding number of children, having a child with IDD, and episodes of depression) warrant investigation in future research; it is possible that these patterns were due to idiosyncratic characteristics of the WLS cohort. Juxtaposed against these limitations is the unique opportunity offered by the Wisconsin Longitudinal Study to evaluate a large unselected random sample of an age cohort for purposes of exploring phenotypic effects of *FMR1* CGG repeat number.

To conclude, we return to our question of whether *FMR1* CGG repeat number polymorphism is associated with phenotypic variability in the general population. Our data suggest that variation in CGG repeats in this gene contributes systematically—even if not clinically—to population characteristics, particularly with respect to cognitive and reproductive functioning. This study illustrates how research inspired by a rare genetic condition (such as fragile X syndrome) can lead to insights about genotype-phenotype associations in the general population.

## Data Availability Statement

The datasets analyzed for this study can be found at https://ssc.wisc.edu/wlsresearch/data/. This study was not preregistered.

## Ethics Statement

The studies involving human participants were reviewed and approved by University of Wisconsin—Madison Institutional Review Board. The participants provided their written informed consent to participate in this study.

## Author Contributions

JH and MM designed the study and drafted the manuscript. JH performed the data analysis. MWB and EB-K carried out the assays. LD, MWB, and EB-K provided critical reviews. All authors contributed to and approved the manuscript.

## Conflict of Interest

MM is the Chair of the Scientific Advisory Board of the John Merck Fund. EB-K has received funding from the following, all of which is directed to RUMC in support of rare disease programs and receives no personal funds and has no relevant financial interest in any of the commercial entities listed: Acadia, Alcobra, Anavex, Biogen, BioMarin, Cydan, Fulcrum, GeneTx, GW, Ionis, Lumos, Marinus, Neuren, Neurotrope, Novartis, Orphazyme, Ovid, Roche, Seaside Therapeutics, Ultragenyx, Yamo, and Zynerba to consult on trial design, development strategies, and/or conducting clinical studies in FXS or other NNDs or neurodegenerative disorders; Vtesse/Sucampo/Mallinckrodt Pharmaceuticals to conduct clinical trials in NPC; Asuragen Inc to develop testing standards for FMR1 testing. The remaining authors declare that the research was conducted in the absence of any commercial or financial relationships that could be construed as a potential conflict of interest.

## Publisher's Note

All claims expressed in this article are solely those of the authors and do not necessarily represent those of their affiliated organizations, or those of the publisher, the editors and the reviewers. Any product that may be evaluated in this article, or claim that may be made by its manufacturer, is not guaranteed or endorsed by the publisher.
